# Collection of social determinants of health in the community clinic setting: a cross-sectional study

**DOI:** 10.1186/s12889-018-5453-2

**Published:** 2018-04-24

**Authors:** Sheila V. Kusnoor, Taneya Y. Koonce, Suzanne T. Hurley, Kalonji M. McClellan, Mallory N. Blasingame, Elizabeth T. Frakes, Li-Ching Huang, Marcia I. Epelbaum, Nunzia B. Giuse

**Affiliations:** 1 Center for Knowledge Management, Vanderbilt University Medical Center, 3401 West End, Suite 304, Nashville, TN 37203 USA; 2 Connectus Health, Nashville, TN USA; 3 Center for Quantitative Sciences, Vanderbilt University Medical Center, Nashville, TN USA; 4 Center for Knowledge Management, Department of Biomedical Informatics, Department of Medicine, Vanderbilt University Medical Center, Nashville, TN USA

**Keywords:** Social determinants of health, PRAPARE, Community health clinic

## Abstract

**Background:**

Addressing social and behavioral determinants of health (SBDs) may help improve health outcomes of community clinic patients. This cross-sectional study explored how assessing SBDs can be used to complement health data collection strategies and provide clinicians with a more in-depth understanding of their patients.

**Methods:**

Adult patients, ages 18 and older, at an urban community health care clinic in Tennessee, U.S.A., were asked to complete a questionnaire regarding health status, health history and SBDs while waiting for their clinic appointment. The SBD component included items from the National Academy of Medicine, the Protocol for Responding to and Assessing Patient Assets, Risks, and Experiences instrument, and the Survey of Household Economics and Decisionmaking. Data collection and analysis occurred in 2017.

**Results:**

One hundred participants completed the study. The questionnaire took approximately 11 min to complete, and the response rate was 90% or higher for all items except annual household income (unanswered by 40 participants). The median number of negative SBDs was 4 (IQR 2.75–7.0), 96 participants had at least one unmet need, and the most common negative SBD was physical activity (75%; 75/100).

**Conclusions:**

The hybrid questionnaire provided insight into a community clinic population’s SBDs and allowed for a more complete understanding than a single questionnaire alone. The brief questionnaire administration time and low non-response rate support the questionnaire’s feasibility in the community clinic setting, and results can be used by clinicians to further the personalization goals of precision medicine. Next steps include evaluating how to connect patients with appropriate resources for addressing their SBDs.

## Background

Community health centers provide care for low-income, medically underserved populations; these individuals are at greater risk of preventable, chronic disease and often report higher rates of chronic conditions, such as hypertension and diabetes [1, 2]. Understanding the role of social and behavioral determinants on health is particularly important for the care of community clinic patients and involves evaluating the impact of upstream factors affecting health, such as education, financial strain, and physical activity. Identifying and addressing social and behavioral determinants in the community clinic setting may represent one strategy for improving health outcomes of this population.

Standardized questionnaires have been developed to better understand patients’ social and behavioral needs. The National Academy of Medicine (NAM) formed a committee in 2013 which recommended a set of measures drawn from validated instruments for systematically collecting information about social and behavioral determinants of health (SBD) for incorporation into electronic health records in the United States [3–5]. The NAM instrument includes the following domains: race and ethnicity, education, financial resource strain, stress, depression, physical activity, tobacco use, alcohol use, social connection or isolation, intimate partner violence, residential address, and census-tract median income. The questionnaire’s performance was tested in two studies, in which the instrument was administered online to participants from across the United States [6, 7]. The studies supported the feasibility, reliability and validity of administering the NAM questionnaire based on the brief completion time (5 min), low non-response rate, lack of question order effects, establishment of test re-test reliability, and observations that response patterns were consistent with the literature and the measures were associated with self-reported physical and mental health [6, 7].

The Protocol for Responding to and Assessing Patient Assets, Risks, and Experiences (PRAPARE) is an instrument developed by the National Association of Community Health Centers and partners for use among United States community health care clinic populations that was designed to align with national social determinants of health initiatives [8, 9]. The PRAPARE instrument covers all domains included in the NAM questionnaire except depression, physical activity, and tobacco and alcohol use. It also includes additional domains not covered in the NAM questionnaire, including farmworker, veteran, housing, insurance, and refugee status, language preference, employment, transportation, neighborhood characteristics, material security (e.g., food, utilities, and clothing) [8] and incarceration history. When possible, questions included in PRAPARE were obtained from validated instruments [9]. Pilot studies were conducted in community health center networks in Hawaii, Iowa, New York, and Oregon, and cognitive testing was used to verify understanding of questions and ease of use [10–12]. Although the PRAPARE questionnaire has not yet been widely implemented in community clinics across the United States, questionnaire developers are currently engaged in implementation plans to facilitate its increased use [12, 13].

While previous studies have begun to assess social determinants of health in the community clinic setting [14, 15], we have not identified any published studies evaluating questionnaire performance in this population using standardized questionnaires, such as the NAM or PRAPARE questionnaires. Systematically capturing information about SBDs will be important to achieve the personalized care goals of precision medicine and help patients develop ways of overcoming upstream factors influencing their health.

In this study, we partnered with a local community health clinic to better understand the performance of a questionnaire for assessing patients’ social and behavioral needs. A hybrid questionnaire approach was chosen in order to gain a more complete understanding of patients’ social and behavioral needs than could be obtained from any single instrument. The questionnaire included all items from the PRAPARE, and items from the NAM SBD questionnaire that are not assessed in PRAPARE (depression, physical activity, and tobacco and alcohol use). Additionally, two items from the Federal Reserve Board’s Survey of Household Economics and Decisionmaking (SHED) were added to gain a better understanding of financial strain [16]. The SHED survey has been administered annually by the Federal Reserve Board since 2013 to a nationally representative adult population across the United States to ascertain the financial and economic status of the country. The primary objective of the study was to evaluate the feasibility of collecting social determinants of health data in the community clinic setting using the hybrid questionnaire approach and assess the prevalence of negative social and behavioral determinants of health.

## Methods

### Study setting and participants

This cross-sectional study was approved by the Vanderbilt University IRB (#162102) and took place at Connectus Health Vine Hill, a healthcare center serving the underinsured population in Nashville, Tennessee, U.S.A.. Recruitment was conducted from February 20 to March 3, 2017. Study team members recruited patients in the clinic waiting room. Patients aged 18 or older who were able to read and speak English were eligible to enroll and full, written informed consent was obtained prior to beginning study procedures. Patients received a $20 Walmart gift card for their participation. Data was collected via paper survey and entered into REDCap [17].

### Study questionnaire

The study questionnaire was administered in the clinic waiting room. The health history and self-rated health status questions were adapted from the Centers for Disease Control and Prevention’s 2017 Behavioral Risk Factor Surveillance System Survey [18]. Participants were also asked to provide their age and gender identity.

The SBD component included all items from PRAPARE, including the optional questions regarding safety, domestic violence, incarceration history, and refugee status. Minor changes were made to a few of the questions for uniformity, clarity, and to aid understanding for individuals with limited health literacy. The changes included spelling out abbreviations, adding the text “please write” prior to the response line for the item regarding number of household members, and removing the text, “This information will help us determine if you are eligible for any benefits” from the item regarding income since it was not applicable for the study. For the social support question, which asks respondents to indicate the frequency of their interactions with people they care about, the response option “5 or more times a week” was replaced with “6 or more times a week” so that response options would not overlap. Yes/no checkboxes for the “other” option regarding material needs were removed since they were not applicable. The text “please write” was replaced with “please describe” prior to the open response options regarding employment and material needs to provide further clarity.

We also included items recommended by NAM that were not addressed in the PRAPARE tool, specifically, depression, physical activity, and tobacco and alcohol use [3]. The questions were added to ensure that information from all actionable domains was captured. To gain insight into participants’ financial strain, we included two questions from the SHED regarding how participants would pay for a hypothetical $400 emergency expense and the largest emergency expense they would be able to pay with money from their checking/savings account [16].

### Statistical analysis

This pilot feasibility study used a convenience sample of one hundred participants with the goal of obtaining a cross-sectional representation of patients seen in the clinic during standard operating hours. All questionnaire responses were examined as outcome variables for the statistical analysis and missing data were excluded. Descriptive statistics were generated from study data using proportions and appropriate measures of central tendency (e.g., means with standard deviations, medians with interquartile ranges).

The depression, physical activity, tobacco use, and alcohol use items from the NAM questionnaire were scored as previously described by the study team [6]. Questionnaire responses about the number of family members and annual household income were used to calculate patient federal poverty levels (FPL) [19]. The following outcomes were included in the classification of negative SBDs: not having housing, housing instability, less than high school education, unemployed and not seeking work, uninsured, at or below 100% FPL, one or more unmet material needs, lack of transportation, social interaction fewer than three times a week, “quite a bit” or “very much” stress, positive depression screening, “inactive” or “insufficiently active” physical activity levels, current smoking status, positive alcohol use screening, inability to pay a $400 emergency with cash or its equivalent, history of incarceration, refugee status, physical or emotional unsafety, and history of domestic violence.

Associations between social determinant domains and age, gender, race, and health history were analyzed using Pearson chi-squared, Wilcoxon rank-sum, and Kruskal-Wallis tests. Spearman rank correlation coefficient and its *p*-value were also conducted to evaluate relationships between questionnaire domains. A two-tailed p-value less than 0.05 was considered significant and correlation strength was considered moderate or higher at ≥ 0.4 or ≤ − 0.4. All analyses were conducted in 2017 using R, version 3.3.2 or STATA/IC, version 12.1.

## Results

We approached 220 patients; 101 enrolled in the study and 119 declined to participate. Of those that declined, 31% (37/119) did not provide a specific reason other than not being interested, 29% (35/119) did not meet study language or age criteria, 23% (27/119) did not have time to complete the questionnaire, 8% (9/119) declined for privacy concerns, 7% (8/119) were not feeling well, and 3% (3/119) were anxious or upset about their clinic visit. The declining population was 22% male (26/119) and 78% female (93/119). One person started the questionnaire, paused for her clinic visit, then withdrew after her visit was over, citing lack of time to complete.

One hundred participants fully completed all study procedures. The study population was 67% female (67/100), 30% male (30/100), and 3% transgender (3/100). The median age of the participants was 38 years (IQR = 26.5–56.5). The two most prevalent self-reported health conditions were hypertension (45%; 45/100) and depression (39%; 39/100). Details on participants’ demographic characteristics, general health, and health histories are in Table 1.

**Table 1:** Participant characteristics and health history

Characteristics	Participants (*n* = 100)
Median age (IQR)	38 (26.5–54.5)
Gender	
Male	30/100 (30%)
Female	67/100 (67%)
Transgender	3/100 (3%)
Ethnicity	
Hispanic or Latino	7/100(7%)
Not Hispanic or Latino	90/100 (93%)
Chose not to answer/Left blank	3/100 (3%)
Race	
Asian only	3/100 (3%)
Pacific Islander only	0/100 (0%)
White only	42/100 (42%)
Native Hawaiian only	0/100 (0%)
Black/African American only	36/100 (36%)
American Indian/Alaska Native only	0/100 (0%)
Other only	7/100 (7%)
Two or more races only	5/100 (5%)
Chose not to answer/Left blank	7/100 (7%)
Farmworker status	
Yes	0/100 (0%)
No	100/100 (100%)
Veteran status	
Yes	6/100 (6%)
No	94/100 (94%)
Language Preference	
English	95/100 (95%)
Language other than English	5/100 (5%)
General Health	
Excellent	14/100 (14%)
Very good	22/100 (22%)
Good	32/100 (32%)
Fair	23/100 (23%)
Poor	7/100 (7%)
Do not know/Not sure	1/100 (1%)
Chose not to answer/Left blank	1/100 (1%)
Health Condition History	
High blood pressure	45/100 (45%)
High cholesterol	28/100 (28%)
Heart attack	3/100 (3%)
Heart disease	10/100 (10%)
Stroke	5/100 (5%)
Asthma	22/100 (22%)
Skin cancer	3/100 (3%)
Other cancer	4/100 (4%)
COPD	14/100 (14%)
Arthritis	30/100 (30%)
Depression	39/100 (39%)
Kidney disease	3/100 (3%)
Diabetes	14/100 (14%)

Data are proportions (%) or medians (interquartile range). *COPD* chronic obstructive pulmonary disease, *IQR* interquartile range

The time to complete the questionnaire and non-response rate were assessed as indicators of feasibility. The final version of the survey contained 36 questions and the median time to complete was 11 min (IQR 8–16). Twenty-two percent (22/100) of participants began the questionnaire, paused for their clinic appointment, and finished it afterwards. With the exception of annual household income, which was left unanswered by 40 participants (40%), the response rate was 90% or higher on all items. Of the respondents that did not provide household income, 78% (31/40) could not afford to pay a $400 emergency with cash or its equivalent.

**Fig. 1:**
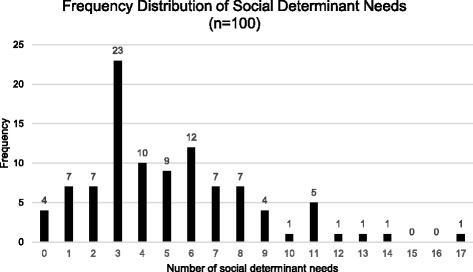
Frequency distribution of social determinant needs

The most prevalent social determinant needs were in the domains of physical activity, financial strain, social integration and support, and stress (Table 2). Seventy-five percent (69/92) reported inactivity or insufficiently active exercise levels, 70% (70/100) were not able to use cash or cash-equivalent to pay for a $400 emergency expense, 45% (25/56) were at or below 100% FPL, 40% (40/100) had fewer than 3 social interactions/week, and 29% (29/100) experienced “quite a bit” or “very much” stress. The median number of negative SBDs (needs) was 4 (IQR 2.75–7.0) and overall, 96% (96/100) of participants had needs in one or more areas (Fig. 1). The total number of needs was significantly correlated with having one or more unmet material needs (*r* = 0.60; *p* < 0.001), an income at or below 100% federal poverty level (*r* = − 0.59; p < 0. 001), positive depression screening (*r* = .57; p < 0.001), appointments affected by lack of transportation (*r* = .46; *p* < 0. 001), and reporting “quite a bit/very much” stress (r = .46; *p* < 0. 001).

**Table 2:** Social determinants of health domains

Domain	Participants (n = 100)
Median number of household family members (IQR) (*n* = 97)	2 (1–3)
Housing Status	
Has housing	87/100 (87%)
Does not have housing	10/100 (10%)
Chose not to answer/Left blank	3/100 (3%)
Housing Stability	
Worried about losing housing	17/100 (17%)
Not worried about losing housing	78/100 (78%)
Chose not to answer/Left blank	5/100 (5%)
Education	
Less than high school degree	15/100 (15%)
High school diploma or GED	37/100 (37%)
More than high school	46/100 (46%)
Chose not to answer/Left blank	2/100 (2%)
Employment	
Unemployed	27/100 (27%)
Part-time or temporary work	24/100 (24%)
Full-time work	26/100 (26%)
Otherwise unemployed but not seeking work	21/100 (21%)
Chose not to answer/Left blank	2/100 (2%)
Insurance	
None/uninsured	20/100 (20%)
Medicaid	28/100 (28%)
CHIP Medicaid	1/100 (1%)
Medicare	20/100 (20%)
Other public insurance (not CHIP)	7/100 (7%)
Other public insurance (CHIP)	2/100 (2%)
Private insurance	20/100 (20%)
Chose not to answer/Left blank	2/100 (2%)
Income	
Median household income (IQR) (*n* = 56)	$19,600 ($9, 897 - $35,750)
Range	$3000 – $105,000
Chose not to answer/Left blank	40/100 (40%)
Invalid response	4 /100 (4%)
Income^a^ (n = 56)	
At or below 100% FPL	25/56 (45%)
Above 100% FPL	31/56 (55%)
Material Security	
Food	18/100 (18%)
Utilities	11/100 (11%)
Medicine or health care (medical, dental, mental health, vision)	27/100 (27%)
Phone	12/100 (12%)
Clothing	12/100 (12%)
Child care	5/100 (5%)
Other	3/100 (3%)
Chose not to answer/Left blank	1/100 (1%)
Transportation	
Lack of transportation affected medical appointments/medicine	15/100 (15%)
Lack of transportation affected non-medical meetings/appointments	10/100 (10%)
Transportation has not affected meetings/appointments	80/100 (80%)
Chose not to answer/Left blank	2/100 (2%)
Social Integration and Support	
Less than once a week	14/100 (14%)
1 or 2 times a week	26/100 (26%)
3 to 5 times a week	22/100 (22%)
More than 5 times a week	34/100 (34%)
Chose not to answer/Left blank	4/100 (4%)
Stress	
Not at all	20/100 (20%)
A little bit	24/100 (24%)
Somewhat	22/100 (22%)
Quite a bit	15/100 (15%)
Very much	14/100 (14%)
Chose not to answer/Left blank	4/100 (4%)
Invalid answer (participant chose more than one response)	1/100 (1%)
Depression (PHQ-2 Score)^a^ (*n* = 88)	
Negative screen (< 3)	67/88 (76%)
Positive screen (≥ 3)	21/88 (24%)
Physical activity (EVS classification)^a^ (*n* = 92)	
Inactive	14/92 (15%)
Insufficiently active	55/92 (60%)
Sufficiently active	23/92 (25%)
Tobacco use	
Never smoker	54/100 (54%)
Former smoker	16/100 (16%)
Current every day or current some day smoker	28/100 (28%)
Unknown if ever smoked	2/100 (2%)
Alcohol use (AUDIT-C score)^a^ (n = 92)	
Negative screen (women < 3, men < 4)	80/92 (87%)
Positive screen (women ≥ 3, men ≥ 4)	12/92 (13%)
Emergency Expense of $400	
Pay with cash or cash equivalent	30/100 (30%)
Pay with non-cash or non-cash equivalent	59/100 (59%)
Pay with combination of cash and non-cash equivalent	11/100 (11%)
Emergency expense of $400^b^ (*n* = 70)	
Would not be able to pay for expense	30/70 (43%)
Credit card, pay over time	12/70 (17%)
Borrowing from a friend or family member	33/70 (47%)
Sell something	10/70 (14%)
Payday loan, deposit advance, or overdraft	7/70 (10%)
Bank loan or line of credit	5/70 (7%)
Other	3/70 (4%)
Largest emergency expense could pay with cash or checking/savings account** (n = 70)	
Under $100	45/70 (64%)
$100 to $199	16/70 (23%)
$200 to $299	2/70 (3%)
$300 to $399	4/70 (6%)
Over $400	2/70 (3%)
Left blank	1/70 (1%)
Incarceration History	
Yes	2/100 (2%)
No	98/100 (98%)
Refugee	
No	98/100 (98%)
Chose not to answer/Left blank	2/100 (2%)
Safety	
Yes	82/100 (82%)
No	11/100 (11%)
Unsure	4/100 (4%)
Chose not to answer/Left blank	3/100 (3%)
Domestic Violence	
Yes	5/100 (5%)
No	91/100 (91%)
Unsure	3/100 (3%)
Chose not to answer/Left blank	1/100 (1%)

Data are proportions (%) or medians (interquartile range). Percentages may not equal 100% due to rounding. ^a^For reporting purposes, the denominators of the Federal Poverty Level, depression, physical activity, and alcohol use domains only consider responses for whom data/scores could be calculated. Reasons for non-calculation of scores include participant non-response, responses that the question was not understood, and responses that the participant did not know the answer to the question. In the case of the AUDIT-C, scores were also not calculated for transgender patients, as the scoring algorithm is gender binary. ^b^For consistency with the Federal Reserve Board’s reporting, this data is shown only for those respondents who indicate they would pay for the expense either in whole or in part using non-cash or its equivalent. AUDIT-C = Alcohol Use Disorders Identification Test, Brief Screen; *CHIP* Children’s Health Insurance Program, *IQR* interquartile range, EVS Exercise Vital Sign, *FPL* Federal Poverty Level, *GED* General Equivalency Diploma, *PHQ-2* Patient Health Questionnaire-2

Several significant associations were identified between health conditions and SBDs. Hypertension was associated with having less than a high school education (*p* < 0.05), unemployment (*p* < 0.05), income at or below 100% FPL (*p* < 0.01), transportation issues (*p* < 0.05), and being unable to use cash or its equivalent to pay for a $400 emergency expense (*p* < 0.05). Those with a history of hypertension also had a higher median number of social needs than those without a history of hypertension (5 vs. 4; *p* < 0.05). A medical history of depression was associated with housing instability (*p* < 0.05), having one or more unmet material needs such as food, medicine, or clothing (*p* < 0.01), having quite a bit or very much stress (*p* < 0.001), and a positive screen for depression on the questionnaire (*p* < 0.01). Individuals with depression also had a higher median number of social needs than those without depression (6 vs. 3; *p* < 0.01).

Respondents’ general health was significantly correlated with age (*r* = − 0.46; *p* < 0.001) and tobacco use (*r* = − 0.41; *p* < 0.001). A few significant, moderate strength or higher correlations were also observed between responses to SBD items; higher stress was correlated with having a positive screening for depression (*r* = 0.51; p < 0.001), a positive screening for depression was correlated with having one or more unmet material security needs (*r* = 0.42; *p* < 0.001), and lower income levels were correlated to lower physical and emotional safety in the respondent’s current home environment (*r* = 0.41; *p* < 0.001).

Study team observation and direct patient interaction provided an opportunity to understand which questionnaire items participants may have had difficulty understanding or answering. For example, on the question asking about annual household income, 11/60 (18%) of participants responded with monthly rather than annual amounts. In response to the insurance question, three respondents wanted to provide more than one answer and five had difficulty classifying their insurance in one of the available categories (e.g., not knowing that their TennCare insurance is Tennessee’s Medicaid program). On the questionnaire item asking how many family members live in the household, five participants initially answered without counting themselves though the question instructions explicitly states to do so.

## Discussion

As rates of chronic illnesses rise across the nation [20], it is important to recognize the role of contributing social and behavioral factors and develop strategies to overcome them. Despite the availability of standardized measures for assessing social determinants of health, SBDs are still not routinely collected in medical practice. Responding to SBDs may help lessen the burden of chronic disease, resulting in improvements in quality of life, increased productivity, and lower health care costs [20, 21]. In states such as Tennessee, where our study was conducted, which have a high prevalence of chronic diseases, including hypertension, diabetes, and cardiovascular disease, addressing SBDs may have a profound impact on overall patient health and on healthcare services utilization [20, 22]. Rates of social and behavioral factors, including smoking, poverty, and lower educational attainment, are higher in Tennessee compared to the nation, and addressing SBDs may help improve the health of the state [20]. While extremely important in all medical settings, meeting patients’ social and behavioral health determinant needs becomes especially critical in community health care clinics. Screening for SBDs must occur at regular intervals, as needs will likely change over time. A necessary next step will be to assess how to help connect individuals with appropriate agencies and resources.

Our study supports the feasibility of administering a social determinants of health questionnaire in the community clinic setting. Most participants were able to complete the questionnaire while waiting for their clinic appointment. The time needed to complete the questionnaire was slightly longer than PRAPARE’s approximate nine minutes, reflecting the inclusion of additional questions. The non-response rate was low for most questionnaire items, with the exception of annual household income.

Responses to the general health questionnaire were consistent with previously-reported data from community clinic populations [23]. The most prevalent negative SBDs were physical activity, financial strain, social integration and support, and stress. Widespread use of standardized SBD questionnaires will enable a better understanding of differences in negative SBDs among different populations. The finding regarding a high prevalence of financial strain was expected, given that the clinic cares for a medically underserved population, where many individuals fall below the federal poverty level [2]. The low rate of alcohol use may reflect the high prevalence of women visiting the clinic for pregnancy-related care in our sample. Associations between health conditions and negative SBDs and associations between SBD domains were also consistent with the literature, further supporting use of the hybrid questionnaire [6, 7, 24].

Results from this pilot study suggest that some questions may need modifications before wide implementation. The question regarding annual household income was unanswered by 40% of participants. Of participants who responded to the question, 20% provided monthly rather than annual amounts. Based on the high non-response rate and the difficulty with answering the question, an alternative approach to understanding financial strain may be needed. Census-tract median income has been used as a way to obtain neighborhood-level information and overcome the sensitivity of asking about household income [3]. However, one of the limitations of this approach is that it may not adequately represent the characteristics of the individual. Additionally, racial minorities and individuals with low incomes are often underrepresented in the U.S. Census, which further compromises reliance on this data for understanding characteristics of these populations [25–27]. As recently as November 2017, the *Huffington Post* reported on the potential negative impact of inaccurate census data on vulnerable communities [28]. The U.S. Census underrepresentation is strikingly apparent when looking at a geographic visualization of “hard-to-count” communities in Nashville, as census-tract areas with higher populations of racial minorities and low-income households display the lowest 2010 census return rates [29]. Another option for assessing financial strain at the individual level is the use of the selected questions from the SHED (Survey of Household Economics and Decisionmaking), for which we observed a high response rate.

The questions regarding insurance and household composition used in our questionnaire may also need refinement. A few participants wanted to select more than one answer to the question regarding insurance, and several had difficulty determining to which category their insurance applied. For the question regarding the number of family members living in the household, a few participants did not include themselves in the count, although the question explicitly indicates to do so, suggesting that the instructions may need to be made clearer.

Limitations of our study include the use of self-reported data and reliance on a convenience sample recruited from a single, urban community health clinic. The majority of participants who enrolled in the study were female. Future studies should aim for a nationally representative demographic sample. While our specific findings regarding the prevalence and type of negative SBDs may not be generalizable to clinics across the United States, our study confirms the feasibility of administering the questionnaire in the community clinic setting. Additional studies are needed to evaluate the impact of questionnaire administration on clinic workflow and incorporation into the electronic health record. LOINC codes have been developed to represent SBD variables; however, a recent 3-year retrospective review of medical records of more than 1 million patients in the nation’s largest health information exchange network found that the social determinant LOINC codes were not being used [30, 31].

## Conclusion

This study supports the feasibility of assessing social and behavioral determinants of health in the community clinic setting. By screening for social determinants of health, providers can obtain a better glimpse of patients’ underlying needs and factors affecting their health. Routine screening for social determinants of health will aid in the implementation of precision medicine, which goes beyond an understanding of a person’s genetic composition to incorporate each patient’s environment and lifestyle to develop individualized treatments. The hybrid questionnaire used in our study provides a more complete view of the patient than through any single instrument. The implementation of the questionnaire will provide insight into a wide range of patient social and behavioral health determinant needs, which will quickly inform providers about the most salient personal and socioeconomic issues of each patient and which domains may require additional probing. Our study has helped reveal top need areas among the community clinic patients that were surveyed, and future studies are planned to evaluate how to connect patients with community and public health resources to help address identified needs.

## Abbreviations

FPL: federal poverty level
IQR: interquartile range
LOINC: Logical Observation Identifiers Names and Codes
NAM: National Academy of Medicine
PRAPARE: Protocol for Responding to and Assessing Patient Assets, Risks, and Experiences
SBD: social and behavioral determinants of health
SHED: Survey of Household Economics and Decisionmaking
